# Intubation learning curve: comparison between video and direct laryngoscopy by inexperienced students

**Published:** 2015

**Authors:** H Aghamohammadi, N Massoudi, M Fathi, A Jaffari, B Gharaei, A Moshki

**Affiliations:** * Anesthesiology Department, Labaffinejad Hospital, Pasdaran, Shahid Beheshti University of Medical Sciences, Tehran, Iran; ** Anesthesiology Research Center, Shahid Beheshti University of Medical Sciences, Anesthesiology Department, Modarres Hospital, Shahid Beheshti University of Medical Sciences, Tehran, Iran

**Keywords:** GlideScope, intubation, simulation, Video Laryngoscopy

## Abstract

**Background:** Direct laryngoscopy (DL) is considered the most common method of tracheal intubation. On the other hand, evidence shows the growing role of video laryngoscopy in danger airway administration.

**Objectives:** Due to the importance of a proper training to accomplish an accurate and fast intubation by the student of anesthesia, this research was conducted to assess the effects of DL and video laryngoscopy (Glidescope VL) training on the success rate of tracheal intubation by low-skill students.

**Materials/Patients and styles:** 50 undergraduate students of anesthesiology took part in this randomized control educational intervention. Having no considerable experience in intubation, they were selected and divided randomly into two equal groups (n = 25); video-laryngoscopy via GlideScope VL and direct laryngoscopy (DL) via a Macintosh blade were prepared by the same experienced anesthesiologist. All the participants practiced intubation six times on the same mannequin within a routine airway situation. The maximum acceptable time for each intubation was 3 minutes and three times of successful intubation was considered as an appropriate intubation skill. The required time for laryngoscopy and intubation at each stage, the grade of glottis view, the reasons for an unsuccessful intubation and the amount of successful intubations were recorded and compared between groups.

**Results:** There was a clear variation between the 2 teams, in all the steps, based on the required time for laryngoscopy and intubation (p = 0.0001). Data analysis was performed by using repeated measures data which demonstrated that the necessary time for laryngoscopy and intubation during the study was clearly lower in the GlideScope VL team (p = .0001). In first five rounds of training, the glottis view in the DL group was significantly better than in the VL group (p < 0.05).

**Conclusion:** Based on the result of today’ study, routine airway intubation by using GlideScope VL is significantly faster than direct laryngoscopy. It seems that further studies are needed to investigate the effect of the educational program on different laryngoscopy and intubation situations.

## Introduction

Direct laryngoscopy is identified as the most common method of tracheal intubation [**1**]. The evidence shows that the advance degree of the Macintosh and Miller leaves are more than 95% of the experienced professionals [**2**,**3**] but in severely ill patients undergoing Urgent Endotracheal Intubation (UEI), the advance degree in the initial try is between 54% and 94%. This will depend on some factors such as emergency situation, unexpected problems, and performer’s experience [**2**,**4**,**5**]. The incidence of particular difficulties such as hypotension (26%), hypoxia (25 %) and death (3%) in UEI is higher than those emerging in the surgery room [**6**,**7**]. So, teaching tracheal intubation and obtaining more skills along with the improvement of intubation instruments seem accurate and may the improve intubation quality and reduce its complications. Currently, there is no standard training method for teaching the beginners and students studying anesthesia [**8**].

GlideScope video laryngoscope, a growing intubation instrument, was introduced in 2001, and provides a better glottic visualization during intubation in the operating room [**1**-**3**], especially in cases via latent or simulated complex airways [**3**]. In his research, Sales concluded that GlideScope VL has a higher overall advance score and a less amount of esophageal problems. He emphasized in emergency intubations that GlideScope VL contributes to an attractive choice to advance initial-try victory for airway administration [**9**].

**Goal**

The objective of the this research was to compare the efficacy of our educational program for intubation training of direct laryngoscopy and GlideScope VL on the advance score of tracheal cannulation in inexperienced pupils.

## Materials/Participants and methods

50 undergraduate students of anesthesiology from Shahid Labbafinejad Hospital, Tehran, Iran took part in this randomized control educational intervention. They had no considerable experience in laryngoscopy and intubation, being elected as candidates to perform this procedure on mannequins. Age, gender, and history of previous intubation training were recorded for all the preachers. Afterwards, they were randomly separated into two equal groups (n = 25). For randomization, a list of all volunteers of 1-50 was made, including even numbers for first and odd numbers for the second group. The first group was taught direct laryngoscopy (DL) with a Macintosh and the second group was taught laryngoscopy with GlideScope VL (Portable, reusable blade video laryngoscope size 4, Vernon Co.) by the same experienced anesthesiologist. 

During the performance of laryngoscopy on mannequins (Laerdal® Airway Management Trainer, normal airway mannequin), the intubation method, and the causality of unsuccessful intubation was investigated and recorded by optic fiber bronchoscopy. Intubation was performed under direct vision in the second group due to using GlideScope VL.

The students in each cluster, practiced intubation six times on the same mannequin. The maximum acceptable time for each intubation was 3 minutes and three periods of successful intubation were considered an appropriate intubation skill. O2 therapy using face mask was taught in prolonged intubation. The required time for laryngoscopy and intubation at each stage, the grade of glottis view (Cormack-Lehane classification of laryngeal view 10), the reasons of unsuccessful intubation and the number of successful intubations were recorded and compared between groups. 

All the gathered data were analyzed by SPSS software version 12, using the chi-square and Fisher’s exact procedure and t-test for the qualitative and quantitative data, respectively. To assess the quantitative data at different times, the repeated ANOVA measures were used. A P factor < 0.05 was proposed as statistically clear.

## Results

50 volunteer undergraduate students of anesthesiology with a mean age of 21.06 ± 0.42 years were assessed in this randomized control educational intervention. There was no statistical distinction between the 2 teams in demographic characteristics and considerable experiences in laryngoscopy and intubation (p > 0.05). 

As summarized in **Table 1**, there was a clear distinction between the 2 teams based on the required time for laryngoscopy and intubation in all stages, while in GlideScope VL group they were significantly lower compared to direct laryngoscopy.

**Table 1:** The mean time required for laryngoscopy and intubation and the mean score for glottis view in the two groups

variable	Laryngoscopy time			Intubation time			Glottis view		
group	DL	VL	P value	DL	VL	P value	DL	VL	P value
1st attempt	121.3 ± 8.6	96.8 ± 5.6	0.0001*	149.4 ± 11.39	110.40 ± 6.75	0.0001*	3.1 ± 0.49	2.6 ± 0.51	0.001*
2nd attempt	108.2 ± 12.8	93.6 ± 9.9	0.0001*	125.08 ± 11.25	106.8 ± 9.0	0.0001*	3.1 ± 0.86	1.8 ± 0.66	0.0001*
3rd attempt	101.2 ± 15.9	84 ± 7.1	0.001*	116.0 ± 12.90	96.0 ± 9.12	0.0001*	2.0 ± 0.20	1.5 ± 0.5	0.0001*
4th attempt	82.5 ± 13.5	69.6 ± 9.8	0.0001*	98.76 ± 8.59	85.24 ± 7.17	0.0001*	2.2 ± 1.85	1.4 ± 0.49	0.026*
5th attempt	65.8 ± 9.5	50.8 ± 9.5	0.0001*	82.28 ± 12.67	66.40 ± 10.75	0.0001*	1.7 ± 0.46	1.2 ± 0.37	0.0001*
6th attempt	38.2 ± 3	30.8 ± 5.7	0.0001*	57.5 ± 6.04	42.32 ± 8.82	0.0001*	1.1 ± 0.3	1.0 ± 0.0	0.077
DL: direct laryngoscopy; VL: GlideScope video laryngoscopy; * statistically significant									

To assess the two variables of laryngoscopy and intubation time trend during the six steps, the analysis of variance of the repeated measures data was used. Intra-group data analysis by Wilks’ lambda was indicative of a significant reduction in the laryngoscopy time in both groups during the study (P: 0.0001, F: 9.84). Also, the intra-group analysis revealed that there was a clear variation among the 2 teams in required time for laryngoscopy for the total of 6 stages (P: 0.0001, F: 4.64).

The intragroup analysis showed that there was a significant increase of performance in both groups during the study (P: 0.0001, F: 13.98). A further analysis revealed a notable distinction between the 2 teams regarding the required time for intubation (P: 0.0001, F: 13.04).

In the first attempt, there was no successful intubation in both groups. In the second attempt, two individuals in the DL group were successful. In both groups, 12 people were satisfying in the third attempt, and the rest could be successful in intubation in the 4th round (p = 0/ 338). The details regarding the reasons for failure in laryngoscopy in the first three stages were summarized in **Table 2**; there was no clear distinction between the 2 teams.

**Table 2:** Causes of unsuccessful laryngoscopy in the two groups

Variable		Inappropriate tongue position	Blade outside vallecula	Laryngoscope loss in the posterior larynx	P-value
1st attempt	DL	18 (72)	7 (28)	-	0.066
	VL	23 (92)	2 (8)	-	
2nd attempt	DL	11 (44)	11 (44)	1 (4)	0.08
	VL	4 (16)	12 (48)	9 (36)	
3rd attempt	DL	3 (12)	11 (44)	1 (4)	0.141
	VL	-	10 (40)	3 (12)	
VL: GlideScope video laryngoscopy; DL: direct laryngoscopy					

## Discussion

The outcomes of the present study demonstrated that GlideScope video laryngoscope could significantly reduce the mean required time for laryngoscopy and intubation in inexperienced students.

Endotracheal intubation is an ideal way to maintain an open airway, to facilitate manufacturing air-conditioning and to stop the occurrence of airway difficulty and aspiration in unconscious patients. Several studies have shown that early airway intubation may improve outcomes in critically ill patients [**11**,**12**]. Therefore, intubation training, as a lifesaving maneuver for health care providers who are in charge of the patient’s health in the emergency department and ICU is essential. 

Traditional methods, direct laryngoscopy using Macintosh and Miller blade, have instrumental limitations [**13**].

In recent years, advances in medical instruments have facilitated tracheal intubation by using video laryngoscopy. A common feature of these tools is the proper glottic view by indirect mechanisms, which does not need to line the mouth, throat, and tracheal route [**14**].

In the present study, both groups had a similar success rate of laryngoscopy. In the study of Silverberg, the success rate in the GlideScope VL group was 15% better than that of direct laryngoscopy group, which was significant [**10**]. In his study, Mosier showed that GlideScope VL can increase the advance ratio of the initial try and may improve the success rate of intubation [**15**]. Of course, the mentioned studies were conducted on people with previous skills of intubation.

In an investigation carried out by Narang et al., residents and professors of an emergency department performed laryngoscopy with a GlideScope and Macintosh blade on a mannequin in a typical situation, fixed neck, and tongue edema [**16**]. The results demonstrated that both at a reasonable position and fixed cervical spine, the participants managed to perform a successful intubation by using Macintosh blade much more quickly compared to GlideScope (p = 0.01), while in the case of tongue edema, the GlideScope group were able to significantly reduce the required time for intubation (p < 0.0001). In the research of Kim et al., 25 emergency specialists were requested to perform intubation by using a GlideScope and Macintosh blade [**17**]. The simulation was implemented in the four states of the normal situation, fixed neck, tongue edema, and a combination of set neck and tongue edema. The findings showed no clear distinction between the 2 teams based on the required time for intubation in the four states, which was different from our result and it could be due to their previous experience.

Furthermore, the results of the review article of Vanderbilt et al., showed that the simulation-based practicing is a very effective way for training GlideScope VL skills [**18**]. Another study was conducted to review 11 educational studies, and found that the use of GlideScope VL could cause a higher percentage of success, faster response rate, and a decreased number of tries to be performed.

The present study showed a normal intubation situation. We did not assess our educational program in difficult intubation situations, which was our study limitation. Also, the intubation complications were not recorded, which was another study limitation.

## Conclusion

Based on the result of the present study, routine airway intubation by using GlideScope VL proved significantly faster than direct laryngoscopy. It seems that further studies are needed to investigate the effect of the educational program on different laryngoscopy and intubation situations.
